# Molecular and Cellular Mechanisms Underlying Somatostatin-Based Signaling in Two Model Neural Networks, the Retina and the Hippocampus

**DOI:** 10.3390/ijms20102506

**Published:** 2019-05-21

**Authors:** Maurizio Cammalleri, Paola Bagnoli, Albertino Bigiani

**Affiliations:** 1 Dipartimento di Biologia, Università di Pisa, 56127 Pisa, Italy; maurizio.cammalleri@unipi.it (M.C.); paola.bagnoli@unipi.it (P.B.); 2 Dipartimento di Scienze Biomediche, Metaboliche e Neuroscienze, Università di Modena e Reggio Emilia, 41125 Modena, Italy

**Keywords:** somatostatin, retina, hippocampus, ion channels, neurotransmitter release, neuromodulation, information flow, network activity

## Abstract

Neural inhibition plays a key role in determining the specific computational tasks of different brain circuitries. This functional “braking” activity is provided by inhibitory interneurons that use different neurochemicals for signaling. One of these substances, somatostatin, is found in several neural networks, raising questions about the significance of its widespread occurrence and usage. Here, we address this issue by analyzing the somatostatinergic system in two regions of the central nervous system: the retina and the hippocampus. By comparing the available information on these structures, we identify common motifs in the action of somatostatin that may explain its involvement in such diverse circuitries. The emerging concept is that somatostatin-based signaling, through conserved molecular and cellular mechanisms, allows neural networks to operate correctly.

## 1. Introduction

Over 40 years after its discovery as hypothalamic peptide inhibiting growth hormone release [1], somatostatin—also known as somatotropin release-inhibiting factor (SRIF)—has been demonstrated to exist in several tissues and to mediate a variety of actions [2]. Somatostatin is widely distributed in the mammalian brain, and somatostatinergic interneurons are recognized as important modulators of neuronal activity [3,4,5,6,7,8,9,10]. The widespread occurrence of somatostatin-based signaling in different neural networks suggests that there might be general and shared mechanisms underlying somatostatin action. To test this hypothesis, in this review, we analyze the properties of the somatostatinergic system in two model networks: the retina and the hippocampus. These nervous structures deal with different and highly specialized computational tasks. Nonetheless, we show that somatostatin acts through common molecular and cellular mechanisms to control information flow across the neuronal circuitries. Moreover, the available data suggest that somatostatinergic systems play key roles in assuring network stability so that neurons can operate correctly.

## 2. An Overview of Retinal and Hippocampal Circuitries

The organization of both retinal and hippocampal circuitries in terms of connectivity and neurotransmission are well known, and here, we summarize their main features in order to better understand the mechanisms of somatostatin action described later. Readers may refer to excellent published reviews for more detailed information (e.g., retina: [11,12,13,14]; hippocampus: [15,16,17,18]).

### 2.1. Retina

**Figure 1:**
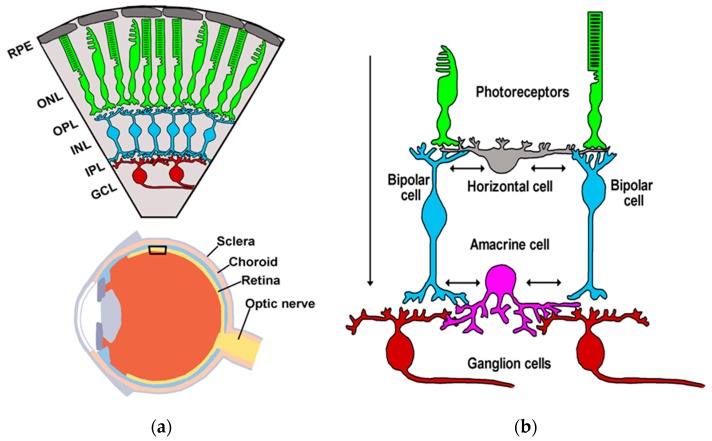
Retinal circuitry: (**a**) Scheme of the eye and sectional view of the layered retina structure. Only the three main classes of principal neurons—photoreceptors (green), bipolar cells (light blue), and ganglion cells (red)—are shown. RPE, retinal pigment epithelium; ONL, outer nuclear layer; OPL, outer plexiform layer; INL, inner nuclear layer; IPL, inner plexiform layer; GCL, ganglion cell layer. (**b**) Schematic drawing of the information flow in retina. Cone and rod photoreceptors send signals to bipolar cells, which, in turn, affect the activity of ganglion cells. The axons of ganglion cells form the optic nerve. Horizontal cells (gray) and amacrine cells (purple) modulate signal transfer across synaptic stations (OPL and IPL). The arrows indicate the vertical and lateral information flows.

In 1893, Ramón y Cajal considered the retina to be “a true nervous center, a peripheral extension of the central nervous system” and stated that “a study of the retina would shed light on the general problem of the connection and mechanism of action of nervous cells” [19]. Since then, the retina has been used extensively as a model of the central nervous system (CNS) for three main reasons [20]: during embryogenesis, it derives from the diencephalon, that is, the retina is part of the CNS; due its location, it is readily accessible for experimental studies; it displays an ordered, layered structure with five basic types of neurons interconnected in a well-defined circuitry (e.g., [21,22]). Retinal neurons include (Figure 1) photoreceptors (the light sensitive cells: rods and cones), bipolar cells, and ganglion cells (the output neurons), which represent the principal neurons, and horizontal and amacrine cells, which constitute the inhibitory interneurons. These cells are organized into clearly distinct layers, three of which house nerve cell bodies (outer nuclear layer—ONL; inner nuclear layer—INL; ganglion cell layer—GCL), and the other two contain synaptic contacts (outer plexiform layer—OPL; inner plexiform layer—IPL) (Figure 1a). The ONL contains cell bodies of rods and cones; the INL contains cell bodies of bipolar, horizontal, and amacrine cells; and the GCL contains cell bodies of RGCs and “displaced” amacrine cells. In the OPL, photoreceptors make synaptic contacts with vertically running bipolar cells and with transversally oriented horizontal cells (Figure 1b). The second synaptic area, IPL, works simultaneously as a relay point between bipolar cells and ganglion cells, and as a station for information processing, which is mainly carried out by amacrine cells (Figure 1b). The basic circuitry within the retina directs the flow of visual information along the pathway, as follows (Figure 1b): photoreceptor → bipolar cell → ganglion cell (vertical information flow). Two horizontal pathways modulate this flow (Figure 1b): one provided by horizontal cells in the outer retina, the other formed by amacrine cells in the inner retina (lateral information flow) [12,23]. Photoreceptors, bipolar cells, and horizontal cells are nonspiking neurons, although they possess a variety of voltage-gated ion channels (see Section 4.1). It is worth noting, however, that some cone bipolar cells may express voltage-gated sodium channels (Na_V_), which seem to be localized in the soma and dendrites [24,25,26,27]. It is believed that these channels may boost subthreshold depolarizing potentials and therefore facilitate electrotonic spread to synaptic terminals [28], or they may signal dark to light transitions [29]. On the contrary, amacrine cells and ganglion cells do generate action potentials.

Retinal chemical synapses play key roles in both vertical and lateral information flow [30]. Glutamate is the excitatory neurotransmitter responsible for the vertical flow of visual signals [31,32]. Glutamate acts on post-synaptic neurons in OPL and IPL through ionotropic (AMPA, kainate, NMDA receptors) or metabotropic glutamate receptors [31,33,34,35]. Lateral information flow is mediated by inhibitory neurotransmitters, such as GABA and glycine, and used by numerous horizontal and amacrine cells to modulate synaptic transmission in the retinal vertical pathway [12,23,32,34]. In particular, GABA is found in horizontal cells and wide-field amacrine cells, whereas glycine is expressed by narrow-field amacrine cells [12,36,37]. As output neurons, ganglion cells integrate excitatory signals from bipolar cells and inhibitory signals from amacrine cells in the IPL before sending visual information to the thalamus. Although glutamate, GABA, and glycine are the main substances that mediate fast point-to-point communication, additional neurotransmitters, including several neuropeptides, are also present in the retina [30,32]. Neuropeptides co-localize with fast-acting neurotransmitters, adding higher complexity to the signaling mechanism, because they typically induce responses with a slower onset and of longer duration [38,39,40,41].

### 2.2. Hippocampus

**Figure 2:**
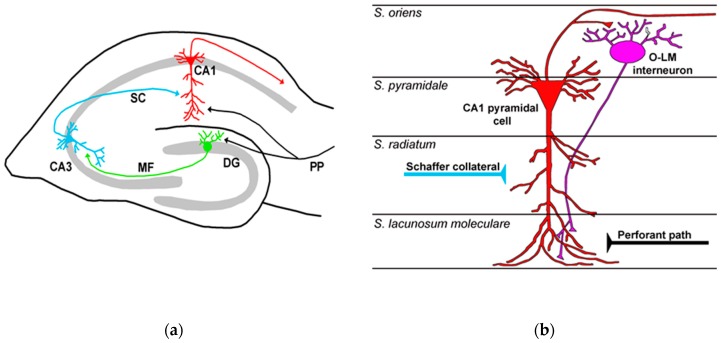
Hippocampal circuitry: (**a**) Signals from the entorhinal cortex (not shown) enter the hippocampus through the perforant pathway (PP) fibers, which make excitatory synapses on CA1 pyramidal neurons (red) and onto granule cells (green) of the dentate gyrus (DG). Granule cells make excitatory synapses via mossy fibers (MF) with CA3 pyramidal cells (light blue). The latter excite CA1 pyramidal cells via the Schaffer collateral (SC) pathway. The axons of CA1 pyramidal cells project to other brain structures, such as the subiculum (not shown). (**b**) Schematic drawing of a CA1 somatostatinergic microcircuit involving an oriens lacunosum-moleculare (O-LM) interneuron. This neuron projects to the stratum lacunosum-moleculare, making synaptic contacts with the distal dendritic tree of pyramidal neurons. O-LM interneuron is mainly activated by local axon collaterals of CA1 pyramidal cells when Schaffer collateral activity drives this cell to its firing threshold.

The hippocampus, located inside brain temporal lobe, has become a model circuit for neuroscientists since the pioneering work on long-term potentiation (LTP) by Bliss and Lømo in 1973 [42]. The hippocampus plays a key role in learning and memory, and receives highly processed sensory information through the entorhinal cortex (EC), which is the gateway to hippocampal circuitry [17,18]. The hippocampus contains both principal neurons (granule cells and pyramidal cells) and several types of interneurons. The regular organization of principal neurons produces a relatively simple layered structure with main synaptic stations in three subfields: the dentate gyrus (DG), cornu ammonis 3 (CA3), and cornu ammonis 1 (CA1) [15,17,43] (Figure 2a). Indeed, the cellular architecture of transverse hippocampal slice is described as an excitatory tri-synaptic circuit. Axons from EC neurons make up the perforant pathway (PP) and terminate on the dendrites of DG granule cells. The axons of these cells (mossy fibers) make synapses on the dendrites of CA3 pyramidal neurons. The latter send their axons (Schaffer collaterals) to the CA1 region and make synapses on CA1 pyramidal neurons, the main output neurons projecting to other brain structures, such as the subiculum. Thus, the hippocampal circuitry directs information flow along the pathway (Figure 2a): DG granule cells → CA3 pyramidal neurons → CA1 pyramidal neurons. Across CA3 and CA1 subfields, the cell bodies of pyramidal neurons are found in the stratum pyramidale (s.p.), and they bear apical (orthogonal) and basal dendritic arborizations (Figure 2b). The apical dendrites have a large shaft extending through the stratum radiatum (s.r.) up to the stratum lacunosum-moleculare (s.l.m.). Basal dendrites, on the contrary, arborize into the stratum oriens (s.o.). It is worth noting that the hippocampal unidirectional mainstream of information flow (Figure 2a) resembles the vertical communication circuit in the retina (Figure 1). In addition to the main tri-synaptic circuit, several local interneuron loops regulate the activity of principal neurons [44,45].

The three main synaptic stations in the hippocampus (DG, CA3, CA1) mediate transmission by using glutamate as an excitatory neurotransmitter. As in retinal circuitry, glutamate activates either ionotropic (AMPA, kainate, NMDA receptors) or metabotropic receptors. AMPA and kainate receptors mediate fast synaptic transmission, whereas NMDA receptors are involved in synaptic plasticity [46], although recent data indicate that calcium-permeable AMPA receptors may also play a role in LTP [47]. Local circuits are driven by GABAergic inhibitory interneurons, which are essential for the appropriate functioning of principal neurons [44]. GABA activates either ionotropic (GABA-A) or metabotropic (GABA-B) receptors. The former mediates fast inhibitory postsynaptic transmission, and the latter mediates slow inhibitory transmission [48,49]. The activity of principal neurons is regulated by a diverse array of inhibitory interneurons, which can be distinguished on the basis of their structural, neurochemical, and electrophysiological features, as well as by the membrane district they make synaptic contacts on [18,44,45,50]. For example, cell bodies and axon hillocks of pyramidal neurons are targeted by parvalbumin- and cholecystochinin-expressing basket cells [51], whereas their dendritic arborizations in the s.l.m. receive synaptic input from somatostatin-expressing horizontal s.o. interneurons [52]. Perisomatic and dendritic inhibition play different but complementary roles in affecting the activity of pyramidal neurons [53].

## 3. The Somatostatinergic System in the Retina and in the Hippocampus

### 3.1. Retina

**Figure 3:**
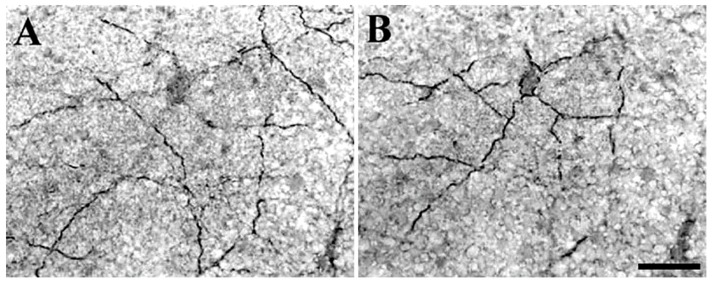
Retinal somatostatinergic interneurons. Photomicrographs of a displaced amacrine cell immunolabelled for somatostatin (avidin-biotin-peroxidase technique) at two depths. Cytoplasmic processes extensively arborize into a meshwork of interwoven varicose fibers at two different levels (**A**,**B**) in the IPL. Scale bar, 30 µm. Modified from [68].

The occurrence of somatostatin in the retina was first demonstrated in extracts of rat retinas by radioimmunoassay and bioassay [54,55]. Subsequently, immunohistochemistry experiments allowed the retinal cells expressing this peptide to be identified. It is now well-established that somatostatin is mainly found in sparsely-occurring, wide-field GABAergic amacrine cells with cell bodies in the INL and by “displaced” amacrine cells with cell bodies located in the GCL (Figure 3) (e.g., [56,57,58,59,60]). Somatostatinergic wide-field amacrine cells are distributed at very low density to all retinal regions, while the population of displaced amacrine cells is confined to the ventral retina. Approximately 5% of GABAergic amacrine cells co-express somatostatin in the larval tiger salamander retina [61]. In cats, less than 0.1% of all amacrine cells show somatostatin-like immunoreactivity [62]. In spite of the very sparse distribution of somata, SRIF-containing arborizations, characterized by multiple varicosities, are extensively distributed throughout the IPL (Figure 3) in all retinal regions (e.g., [59,60]), suggesting a diffuse action of somatostatin in the retinal circuitry. This is further supported by the widespread distribution of somatostatin receptors (see below). It is noteworthy that the majority of synaptic contacts of somatostatinergic amacrine cells occur with processes of other amacrine cells [62]. Although most amacrine cells possess no axon, those containing somatostatin often have long “axon-like” processes, which probably function as true axons [58]. In rodent retina, SRIF-containing amacrine cells, like most amacrines, are under the control of the rod pathway [63]. In chick retina, changes in somatostatin immunoreactivity are driven by the light–dark cycle [64]. However, the functional properties of retinal somatostatinergic interneurons are poorly understood. In particular, it is not known whether they are spontaneously active, as is the case for other amacrine cells, such as dopaminergic ones [65,66]. Recently, major interactions have been determined between SRIF- and dopamine (DA)-releasing amacrine cells in the non-image-forming visual circuit of the mouse retina [67]. In a few species, somatostatin immunoreactivity has also been identified in sparse ganglion cells [59].

Somatostatin produces its effects by activating five heptahelical transmembrane G-protein coupled receptors (GPCRs), named SST_1_–SST_5_ [2]. Somatostatin receptors have been immunohistochemically localized to different retinal cell populations, suggesting that somatostatin may act at multiple levels of the retinal circuitry, and, in particular, in both OPL and IPL [59]. SST_1_ is expressed by SRIF-containing amacrine cells, and it is believed to work as an autoreceptor that regulates somatostatin release [68,69,70,71,72]. SST_1_ is also expressed by some rat ganglion cells [69] and dopaminergic amacrine cells [68]. SST_2_ occurs in two isoforms, SST_2A_ and SST_2B_ [2]. SST_2A_ is abundantly expressed and localized predominantly to cone photoreceptors, rod bipolar cells, horizontal cells, and to specific amacrine cell populations, including dopaminergic and glycinergic ones [59,60,73,74]. The localization of SST_2A_ in the axon terminals of rod bipolar cells suggests that somatostatin may regulate glutamate release (see Section 4.1). In humans, SST_2A_ immunoreactivity has been found in all retinal layers [75]. The SST_2B_ isoform is mainly localized in the membrane of photoreceptors, indicating that somatostatin has activity in the outer retina [76]. No data are available about the occurrence of SST_3_ in the mammalian retina [2,77]. In the newt retina, SST_3_ has been detected in the inner segment of cones and in the connecting cilia of rods, which correspond to the outer photoreceptor segments [78]. This is in agreement with the demonstration that SST_3_ is localized in the neuronal cilia in many brain regions, in which it might play a role as chemical sensor [79] or in ciliary trafficking [80]. SST_4_ is localized in the ganglion cells in both mouse [60,67] and rat [81] retinas. In purified ganglion cell culture, immunolabeling occurs over somata and dendrites [81]. SST_5_ is found in dopaminergic, cholinergic, and GABAergic amacrine cells in the inner retina [82], in horizontal cells of the outer retina [83], and also in a subset of ganglion cells [84]. Somatostatin receptor activation leads to the modulation of different signaling pathways (Section 3.3).

### 3.2. Hippocampus

**Figure 4:**
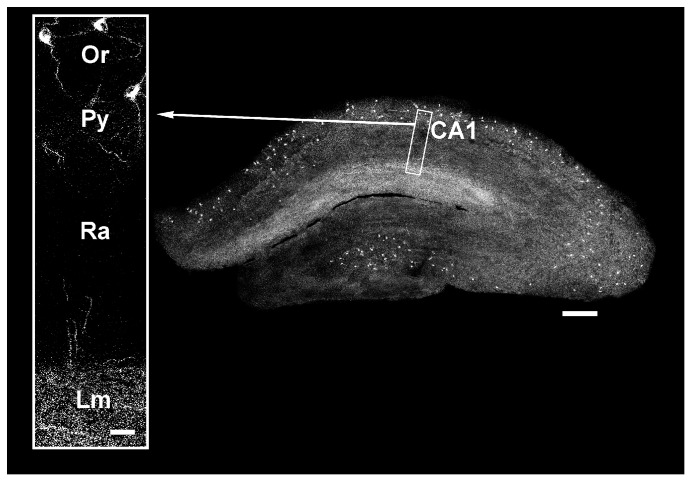
Hippocampal somatostatinergic interneurons. A tissue slice of mouse hippocampus was processed for immunocytochemistry with monoclonal antibody directed against somatostatin and observed with confocal microscopy. The low magnification image is a reconstruction of seven confocal optical images. Note that SRIF-immunoreactive cell bodies (white dots) occur throughout the hippocampal formation. *Inset*: High magnification image of the CA1 subfield. Somata of SRIF-positive neurons can be detected in the s.o. (Or) but not in the s.p. (Py), whereas immunolabelled fibers are found in the s.r. (Ra) with terminal arborizations in the s.l.m. (Lm). Extensive somatostatin labeling is localized to the Lm, which therefore represents the release site. Scale bars: 200 µm for the low magnification image; 20 µm for the inset image. Modified from [99].

The presence of somatostatin in the hippocampus was first demonstrated through a specific radioimmunoassays on rat brain samples [85]. Subsequent immunohistochemistry studies confirmed the occurrence of SRIF-positive neurons in all hippocampal subfields (e.g., [86,87,88,89]). It is now known that somatostatin is expressed by specific subsets of inhibitory interneurons, which also contain GABA (e.g., [90,91]). As recalled above (Section 2.2.), there is an incredible variety of GABAergic interneurons in the hippocampal circuitry [44,45,52]. Somatostatinergic interneurons belong to the subset of dendritic inhibitory interneurons which target the dendritic domains of principal cells. There are two main groups of them in CA1 and CA3 subfields: bistratified cells (BiCs) and oriens lacunosum-moleculare interneurons (O-LMs). These interneurons are most commonly found in electrophysiological studies on tissue slices [52]. Cell bodies of BiCs are located in s.p. and s.o., where their dendritic tree arborizes horizontally, whereas their axon branches in s.o. and s.r., where they target both the basal and apical dendritic districts without synapsing the cell bodies of principal cells. O-LMs are horizontal cells located in the s.o. (Figure 2b). They have dendritic arborizations running horizontally in s.o. and emit an axon extending to s.l.m., where it branches heavily and targets the distal apical dendritic tree of CA1 pyramidal cells (Figure 2b and Figure 4). O-LMs are the most studied hippocampal interneurons, as they drive prototypical feedback inhibition on pyramidal cells and participate in hippocampal theta (6–10 Hz) activity [44,45,52,92]. In CA1, O-LMs represent about 4.5% of all interneurons. An intriguing property of these neurons is their spontaneous activity, which would likely provide a tonic inhibition on pyramidal cells [93,94]. In the CA1 subfield, O-LMs receive major excitatory input from local, recurrent axon collaterals of pyramidal neurons [44,52] (Figure 2b). The O-LM equivalent in DG is the hilar perforant pathway-associated cell [44]. It is noteworthy that somatostatinergic terminals are mainly located at the distal part of the dendrites of DG granule cells and CA3/CA1 pyramidal neurons [95,96] (Figure 4), whereas somatostatin receptors occur throughout the dendritic tree and cell bodies of principal neurons (see below).

With the exception of SST_5_, all of the somatostatin receptor subtypes have been detected in the hippocampus [97,98]. Despite the overlapping distributions, they show a high degree of specialization with regard to their subcellular targeting. In CA1 of the mouse hippocampus, SST_1_ immunoreactivity is found to be associated with cell bodies of pyramidal neurons and with fibers of s.r. and neuropils of s.l.m., indicating pre- and post-synaptic localization [99]. SST_1_ and somatostatin co-localize in somatostatinergic interneurons in s.o., suggesting a role for SST_1_ as an autoreceptor [99]. Indeed, blocking SST_1_ induces a considerable increase in the hippocampal somatostatin level without affecting the GABA level [100]. In rats, SST_1_ occurs in the DG granule cell layer and in the s.p. and s.o. of the CA3 and CA1 subfields [96]. Diffuse SST_2A_ immunolabeling is detected in DG, whereas the CA3 region is negative [96]. In rat CA1 pyramidal neurons, SST_2A_ is associated with cell bodies and basal (s.o.)/apical (s.r.) dendrites, as well as with axon terminals in the s.r. (Schaffer collaterals), suggesting pre- and post-synaptic effect of somatostatin [101]. SST_3_ is expressed in all hippocampal subfields, does not localize to pre- or post-synaptic sites, and is selectively associated with nonmotile neuronal cilia [79,102,103]. The occurrence of SST_3_ in these structures suggests that it may work as a chemical sensor. Interestingly, SST_3_-KO mice show deficits in object recognition memory and impairment in adenylyl cyclase (AC)/cyclic adenosine monophosphate (cAMP)-mediated LTP in the CA1 hippocampus [104]. Finally, SST_4_ immunolabeling is detected in all hippocampal subfields [96]. In rat CA1 pyramidal neurons, SST_4_ occurs almost exclusively on the apical dendrites in s.r. where Schaffer collaterals end and in their somata, suggesting a postsynaptic function [105]. As was nicely pointed out by Qiu et al. [106], these neurons are “virtually coated with somatostatin receptors, suggesting that somatostatin is an important signaling molecule in this region”.

### 3.3. Signal Transduction Mediating Somatostatin Action

Somatostatin acts through its receptors (SSTs) by affecting G-protein-mediated intracellular signaling pathways, which include specific molecular components [2]. Typically, SSTs signal through Gi/Go proteins, heterotrimeric guanine-nucleotide-binding proteins characterized by sensitivity to pertussis toxin (PTX), and inhibitory coupling to adenylyl cyclase (AC). The latter is the enzyme responsible for the production of the second messenger, cAMP, which activates protein kinase A (PKA). Thus, canonical signal transduction by SSTs mediates inhibitory effects on the AC/cAMP/PKA pathway [2]. Components of this signaling pathway occur in retinal and hippocampal neurons (e.g., [107,108,109]), although specific variations have been described. In the mouse retina, for example, activation of SST_1_ or SST_2_ individually does not affect AC activity. However, by blocking either SST_1_ or SST_2_ with specific antagonists, receptor coupling to AC inhibition occurs, suggesting that there may be interactions between SST_1_ and SST_2_ [110]. Further complexity of the signal transduction cascade is provided by modulatory mechanisms that enhance or reduce the effect of SST activation. In the CA1 pyramidal neurons of the mouse hippocampus, Ser/Thr protein phosphatase activity regulates somatostatin signaling [111]. In addition to the AC/cAMP/PKA pathway, other intracellular enzymatic cascades may mediate the effects of somatostatin. In retinal ganglion cells, SST_4_ activation is signaled by Gβγ and protein kinase C (PKC) pathways [112]. In rat hippocampal neurons maintained in culture, prolonged exposure to somatostatin activates G-protein-coupled receptor kinase (GRK) and PKCε [113]. Moreover, somatostatin dampens firing in CA1 pyramidal neurons by downregulating the cyclooxygenase 2 (COX-2)/prostaglandin E_2_ (PGE_2_) pathway in a mouse model of epileptiform bursting [114]. In a nutshell, intracellular pathways driven by SSTs may vary considerably. This diversification is a hallmark of the somatostatin action (pleiotropic effects: [115]). Retinal ganglion cells provide an excellent example of the functional diversification stemming from multiple intracellular signaling. These neurons express both SST_4_ and SST_5_ (Section 3.1.): when activated by somatostatin, SST_4_ signals to voltage-gated Ca^2+^ channels via the Gq-PKC pathway [112], whereas SST_5_ signals to AMPA receptors via Gi/o–cAMP–PKA [84]. An important open issue is the receptor–receptor interactions at the level of either receptor proteins or intracellular signaling. There is increasing evidence for this type of molecular mechanism to explain the modulatory effect of somatostatin in both the retina and hippocampus (e.g., [70,106,116,117]). Dissecting out the molecular physiology of somatostatin receptors will provide new insight into the somatostatin action in neural networks.

### 3.4. The Complexity of Somatostatin-Based Signaling: Autocrine, Paracrine, and Synaptic Communication

Somatostatin is stored in intracellular vesicles and is secreted through a calcium-dependent mechanism [118,119,120]. However, once released, somatostatin is involved in different aspects of cellular communication, encompassing basically all known signaling mechanisms that may occur inside a neural network. Somatostatinergic interneurons express a specific receptor, SST_1_, which regulates somatostatin release [70,71,99,100]. This autocrine mechanism affects the level of somatostatin, as demonstrated in SST_1_-KO mice [70] or by blocking SST_1_ pharmacologically [100]. On presynaptic membranes, somatostatin depresses the release of excitatory neurotransmitters through an effect on voltage-gated calcium channels (see Section 4). In this way, somatostatin influences the synaptic communication between neurons by acting not through the mechanism of synaptic integration, but through the modulation of synaptic transmission mediated by classical neurotransmitters. Somatostatin may also diffuse out of the synaptic space and reach nearby molecular targets in the postsynaptic neuron as well as in adjacent neurons. This paracrine signaling has its best experimental support by the observation that the distribution of SSTs exceeds the somatostatinergic synaptic terminals (see Section 3). In mammalian retina, SSTs occur throughout all layers and are also expressed by photoreceptors and bipolar cells [59,60]. It is noteworthy that the weft of SRIF-containing processes is not densely woven (Figure 3), a structural feature suggesting slow, diffusion-limited activity of somatostatin on retinal neurons [62]. In the hippocampus, somatostatin terminals are mainly located close to the distal dendrites of the principal neurons in DG, CA3, and CA1 [44,95,96]. However, SSTs are also found in other subcellular districts. In CA1, for example, SSTs occur not only in the apical dendritic arborizations but also along the main dendritic shaft and cell bodies of pyramidal neurons as well as on the axon terminals of Schaffer collaterals [101,105]. In other words, there is a mismatch between the location of somatostatin and its receptors, suggesting that released somatostatin must spread over several micrometers to reach SSTs [40]. What is the significance of this paracrine communication? One possibility is that it allows overcoming the structural burden provided by point-to-point synaptic contacts. Indeed, it is likely that somatostatin release occurs mainly in extra-synaptic sites [39,40,121]. Released somatostatin has a half-life of several minutes before being degraded by tissue peptidases (e.g., [122,123]). Thus, it may reach different membrane districts of target cells to drive regulatory actions and to control the activity of large and diverse neuron populations [124] (see Section 4). It is important to recall that in the retina, paracrine signaling is not an exclusive feature of the somatostatinergic system. Upon light stimulation, dopaminergic amacrine cells release GABA and DA extrasynaptically, acting at a distance via diffusion to control the adaptation of the retinal circuitry to light [125]. Since somatostatinergic amacrine cells are driven by the rod pathway, there is an interaction between these two paracrine signaling systems (SRIF and DA) to regulate light adaptation [63].

## 4. Molecular and Cellular Physiology of Somatostatin Action: Modulation of Membrane Excitability and Neurotransmission

Early indications that somatostatin could influence neuron activity came from intracellular recordings with sharp microelectrodes in rat tissue slices (e.g., [126]) and extracellular recordings of single units in the rat brain (e.g., [127]). Later on, the development of the patch-clamp [128] and calcium-imaging [129] techniques allowed researchers to characterize the effects of somatostatin on membrane conductance and intracellular Ca^2+^ signals in a variety of tissues, including the retina and hippocampus. There is now an overwhelming set of data indicating that somatostatin targets specific proteins underlying membrane excitability and neurotransmission. In this way, somatostatin affects two key aspects of neuron functioning, namely the ability to produce electrical signals and the ability to transfer information.

### 4.1. Retina

A reasonably large variety of ion channels are present in retinal cells, where they play a key role in transforming visual cues into specific electrical activity. By modeling the membrane currents of the rod photoreceptor inner segment according to the Hodgkin and Huxley equations, Kamiyama and collaborators (1996) determined that different types of ion channels are involved in shaping the voltage response to light after the initial transduction events [130]. Also, in bipolar cells, membrane electrical responses depend on the interplay of several ion channels [26,131]. In ganglion cells, the output neurons of the retina, ion channels underlie action potential firing, a fundamental process for relaying information to the thalamus. Given the importance of ion channels in the physiology of retinal circuitry, it is not surprising that somatostatin exerts a modulatory action on these membrane proteins, affecting cell activity and signal processing.

**Figure 5:**
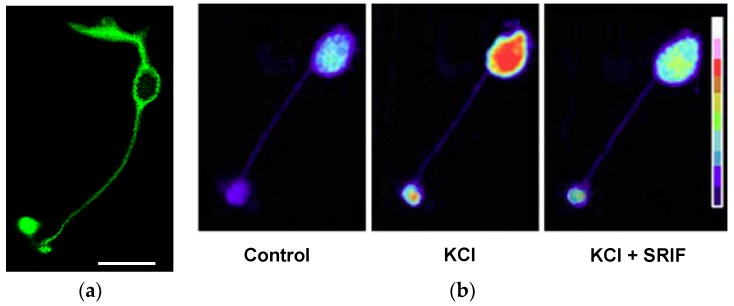
Modulation of Ca^2+^ accumulation by somatostatin via SST_2A_ in isolated rod bipolar cells (RBCs). (**a**) RBCs express the somatostatin receptor SST_2A_. Immunocytochemistry with antibody against the SST_2A_ protein in a mouse RBC. Scale bar: 10 μm. (**b**) Fluorescence, pseudocolor images of a rabbit RBC loaded with fluo-3 to monitor intracellular [Ca^2+^] and observed by confocal laser microscopy. Application of 60 mM KCl to depolarize cell membrane KCl indicated an increase in [Ca^2+^] as compared to its level recorded when the cell was bathed in physiological solution (control). In the presence of KCl, the addition of 200 nM somatostatin (KCl + SRIF) significantly reduced the intracellular level of [Ca^2+^]. The fluorescence (right vertical bar) is an arbitrary 256-point grey scale converted to pseudocolor. Modified from (**a**) [116]; (**b**) [136].

In retinal cells, a common motif of the somatostatin action is the alteration of Ca^2+^ influx through a modulatory effect on voltage-gated calcium channels. Akopian and collaborators (2000) first showed the effects of somatostatin on voltage-gated L-type calcium channels (Ca_V(L)_) in rod and cone photoreceptors of the salamander retina [132]. The effect differs between the two photoreceptor types: in rods, calcium currents are reduced, whereas in cones they are augmented. Consistent with the electrophysiological results, Ca^2+^ accumulation is inhibited in rods and enhanced in cones [132]. Interestingly, the effect of somatostatin on Ca_V(L)_ in cultured chicken cone photoreceptors is dependent on the circadian phase, being inhibitory during the night but not during the day [133]. In bipolar cells isolated from goldfish, somatostatin is able to suppress calcium currents [134]. By analyzing intracellular calcium levels in rod bipolar cells (RBCs) isolated from rat retina, Johnson and collaborators (2001) showed that somatostatin affects calcium influx by exerting an inhibitory effect on Ca_V(L)_ [135]. Also, in rabbit RBCs, calcium-imaging experiments demonstrated that Ca^2+^ accumulation upon membrane depolarization is depressed by inhibiting Ca_V(L)_ through SST_2_ activation [136] (Figure 5). In rat retinal ganglion cells, stimulation of SST_4_ with somatostatin or its specific agonist, L-803,087, reduces L-type Ca^2+^ current [67,81,112].

Somatostatin also affects voltage-gated potassium channels in retinal cells. In both rod and cone photoreceptors of salamander retina, somatostatin potentiates a delayed rectifier K^+^ current (K_V_) but not the Ca^2+^-dependent K^+^ current (K_V(Ca)_) [132]. In rabbit RBCs, on the contrary, the latter was reduced following specific stimulation of SST_2A_ with octreotide, a somatostatin analog [136]. This effect, however, was likely due to a reduction in Ca^2+^ influx, since somatostatin inhibits calcium channels in these neurons [135,136]. Interestingly, in rabbit RBCs, delayed rectifier K^+^ current was not affected by somatostatin [136]. In rat retinal ganglion cells, activation of SST_4_ receptors potentiated a 4-AP-sensitive, voltage-gated K^+^ current [81]. Also, in another subset of ganglion cells, the melanopsin-containing intrinsically photosensitive retinal ganglion cells (M1 ipRGCs), somatostatin, or the SST_4_ agonist, L-803,087, produced an increase in voltage-gated potassium currents and reduced the intrinsic spontaneous firing rate [67].

To modulate ion channel functioning, somatostatin acts via specific G protein-mediated intracellular pathways, which may differ significantly according to the SST expressed in target cells [115]. In photoreceptors of the salamander retina, the effect of SST_2_ activation on K_V_ and Ca_V_(_L_) is blocked by PTX, an inhibitor of Gi/o protein, and reduced by intracellular infusion of GDP-β-S, a non-hydrolysable inhibitor of Gα proteins [132]. On the contrary, modulation of Ca_V(L)_ by SST_4_ activation is not sensitive to PTX but to pharmacological inhibition of the Gβγ complex or PKC in rat retinal ganglion cells [112]. Remarkably, the effects on Gβγ and PKC were additive, suggesting that two distinct intracellular pathways were activated by SST_4_.

What is the biological significance of the modulatory action of somatostatin on ion channels in retinal cells? To answer this question, let us consider the following signaling pathway for which we have detailed information: rod photoreceptor (RP) → rod bipolar cell (RBC) → ganglion cell (GC) (Table 1). This is clearly a simplification, because in mammals, RBCs relay information to ganglion cells indirectly through a specific circuit between RBCs, AII amacrine cells, and cone bipolar cells [21,63]. In addition, there are several parallel information lines underlying coding of visual information in the mammalian retina [137]. For the purpose of our reasoning, however, the analysis of the pathway RP → RBC → GC can give us some interesting hints.

It is clear from Table 1 that whatever the target cell is, somatostatin tends to reduce the activity of Ca_V(L)_, which is considered a key ion channel in retinal processing [138]. The main consequence of this effect is a decrease in Ca^2+^ influx and, therefore, a reduction in the intracellular Ca^2+^ signal. Since in RPs and RBCs, synaptic transmission is a Ca^2+^-dependent process (e.g., [139,140,141]), the expected functional consequence of somatostatin action is a reduction in neurotransmitter release. Consistent with this prediction, glutamate release detected by HPLC is reduced in the presence of somatostatin or its analog, octreotide, which displays a high selectivity for SST_2_ in mouse retinal explants [116,142]. In RBCs, calcium-imaging experiments on specific cell districts showed that somatostatin is able to modulate calcium influx in both axonal terminals and in the cell bodies [135,136]. In RBCs isolated from rat retinas, immunocytochemical observations indicated that SST_2A_ receptor immunostaining localizes to the dendrites, cell bodies, axons, and axon terminals [135]. A similar distribution was also found in RBCs isolated from mice [116] (Figure 5a) and rabbits [136]. As a whole, these observations support the notion that somatostatin, via the SST_2_ receptor, is able to exert modulatory control on the glutamatergic transmission in RBCs. An analogous mechanism has also been proposed for glutamate release from photoreceptors [132]. Less understood is the significance of somatostatin action through its receptors on the dendrites and cell bodies of RBCs. In rat RBCs, Ca_V(L)_ is strategically located at axon terminals to control neurotransmitter release [143,144,145]. However, somatostatin affects the intracellular accumulation of Ca^2+^ in rabbit RBCs in both the terminals and somata [136]. Electrophysiological recordings from rabbit RBCs with cut axons might help to solve this issue (e.g., [145]). The significance of the inhibitory effect on Ca_V(L)_ and intracellular Ca^2+^ signaling in retinal GCs, which are output neurons, is not clear [81]. It is worth noting, however, that M1 ipRGCs act as presynaptic elements for DA amacrine cells (postsynaptic elements) [146].

In rat retinal GCs, immunostaining for SST_4_ is localized to dendrites and cell bodies [81]. Unlike RPs and RBCs, ganglion cells generate fast action potentials to transmit information to the thalamus. It is then conceivable that somatostatin modulatory action on K_V_ (Table 1) may affect firing activity. Indeed, specific stimulation of SST_4_ produces a reduction in the frequency of action potentials induced by the injection of depolarizing current in GCs [81]. Also, somatostatin and the SST_4_ agonist L-803,087 reduce the spike frequency in spontaneously active M1 ipRGCs [67]. To our knowledge, there are no reports of somatostatin acting on voltage-gated sodium channels in retinal GCs. This is an interesting aspect of the somatostatin effect on membrane excitability: the capability of generating rapid impulses is preserved, while neuronal information (that is, series of impulses over time) is modulated by the effects on firing properties, such as the frequency. The significance of the effect on K_Ca_ in RBCs (Table 1) is less clear. According to the modeling of membrane ionic currents proposed by Usui and collaborators (1996) for bipolar cells [131], K_Ca_ is involved, along with other channels, in generating the electrical responses of these cells. Thus, it is conceivable that the reduction of K_Ca_, even if it is a consequence of the effect of somatostatin on Ca_V(L)_ [136], may significantly affect electrical signaling in RBCs. In this regard, it is interesting to recall that bipolar cells in teleost retina are capable of producing Ca^2+^ action potentials [144] and presynaptic Ca^2+^ transients [147] in response to light flashes after dark adaptation, and that Ca^2+^-activated K^+^ current is a key component of spiking activity [148].

Somatostatin also targets neurotransmitter receptors in the transmission line RP → RBC → GC. A patch-clamp recording and calcium-imaging study on a subset of rat retinal GCs showed that somatostatin suppresses AMPA responses via SST_5_ receptors through a rather complex intracellular pathway involving cAMP/PKA and the release of Ca^2+^ from intracellular stores [84]. AMPA receptors are the main non-NMDA receptors responsible for ganglion cell activation by glutamate released by bipolar cells [149].

In addition to principal neurons, somatostatin affects the activity of retinal interneurons. In organotypic retinal slices, somatostatin enhances GABA-A responses in rat amacrine cells, suggesting a possible role in facilitating the GABAergic inhibitory transmission in the retinal circuitry [150]. In cultured rat amacrine cells, on the contrary, activation of SST_5_/SST_2_ receptors reduces GABA through the inhibition of voltage-gated Ca^2+^ channels (L- and N-types) and via negative coupling with cAMP/PKA signaling pathway [151]. PKA downregulation could reduce the phosphorylation of Ca^2+^ channels with consequent reduction in Ca^2+^ influx and GABA release [115]. Somatostatin, through SST_2A_, inhibits both K^+^ and Ca^2+^ channels and spontaneous firing in mouse dopaminergic amacrine cells involved in a specific microcircuitry in the inner retina [67].

In summary, the available data suggest that somatostatin affects the functioning of retinal principal neurons by exerting mainly a dampening effect on information transmission. The goal of this effect is likely to set the correct dynamic range of neuronal activity in the network (see Section 5.3). Indeed, the widespread arborization of somatostatinergic amacrine cells suggests that somatostatin “does not have a role in fine-grain visual information processing in the mammalian retina, but likely modulates the overall retinal circuitry” [59]. In addition, the electrophysiological studies reported above indicate that the effects of somatostatin on the functional properties of retinal cells have a slow onset and often last after its removal (e.g., [132,136]). This slow time course does not match the more rapid processing of visual information [124].

### 4.2. Hippocampus

Early intracellular recordings from CA1 pyramidal neurons in hippocampal slices provided conflicting results as to the action of somatostatin on membrane excitability (e.g., [152,153,154]). Soon after, it became clear that the excitatory effects of somatostatin were actually due to the indirect action on acetylcholine neurotransmission: somatostatin alone hyperpolarizes hippocampal pyramidal neurons and depresses their firing (e.g., [155,156]). It is now generally accepted that somatostatin exerts mainly an inhibitory action on hippocampal principal neurons. Unlike the retina, somatostatinergic interneurons are found throughout all hippocampal subfields. Among them, CA1 region is the best studied in terms of somatostatin effect on membrane excitability and neurotransmission. Therefore, here, we refer mainly to this region.

**Figure 6:**
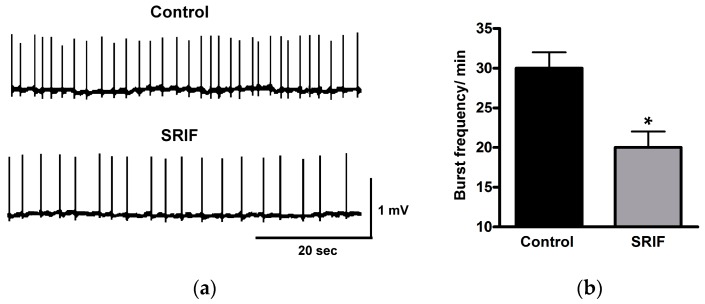
Somatostatin reduces the firing rate of mouse hippocampal neurons. Extracellular recordings with a sharp microelectrode were performed from the stratum pyramidale of the CA1 subfield in a tissue slice. (**a**) Spontaneous firing (Control) was induced by perfusing tissue with Mg^2+^-free medium containing the potassium channel blocker 4-AP. Application of 1 μM somatostatin (SRIF) reduced the firing rate. (**b**) Quantitative analysis for the somatostatin effect shown in (**a**). Somatostatin produced a decrease of ~30% in the frequency of action potentials. * p < 0.05. Modified from [114].

A general mechanism to produce membrane hyperpolarization and to reduce the firing rate is the activation or potentiation of membrane K^+^ currents [157]. Hippocampal pyramidal neurons express several types of these currents [158], and electrophysiological experiments have shown that somatostatin targets a non-inactivating, voltage-dependent current known as the M-current (I_M_) as well as K^+^ currents affecting the resting potential [159,160,161,162]. The M-current (M stands for inhibitory *muscarinic* agonists [158]) counteracts membrane depolarizations and tends to reduce the firing rate by shifting the membrane potential away from the action potential threshold. By using somatostatin receptor knock-out (KO) mice and subtype-specific ligands, it has been possible to demonstrate that the effect on M-current is due to the activation of SST_4_ [106]. As for the intracellular signaling cascade, studies suggest that arachidonic acid metabolites mediate I_M_ potentiation through the activation of phospholipase A_2_ (PLA_2_; [161,163]). The M-current has a limited role in setting the resting potential of hippocampal neurons and therefore does not explain the hyperpolarization induced by somatostatin [153,159]. As demonstrated by Schweitzer et al. (1998), somatostatin activates a voltage-insensitive K^+^ leak current (I_K(L)_) that exerts a stabilizing effect on the resting membrane potential of CA1 pyramidal neurons in rat hippocampal slices [162]. In cultured rat hippocampal pyramidal neurons, a G protein-gated, inwardly rectifying potassium (GIRK) current is also enhanced by somatostatin, whereas it is inhibited by PTX [164]. GIRK is activated by the Gβγ subunit of Gi/o and may help to stabilize the resting membrane potential [165]. Finally, somatostatin induces membrane hyperpolarization and reduces spiking by activating K^+^ leak channels in mouse hippocampal slices [111]. Interestingly, somatostatin also affects firing by stimulating the insertion of pre-formed GIRK channels into the cell membrane [111]. In summary, somatostatin dampens membrane excitability in CA1 pyramidal neurons by targeting distinct potassium channels that lessen membrane depolarization, therefore reducing the firing rate (Figure 6).

In addition to regulating single cell excitability, somatostatin plays a major role in controlling synaptic communication mediated by fast neurotransmitters. In pyramidal neurons freshly dissociated from the rat hippocampal CA1 region, somatostatin reduces voltage-gated N-type calcium currents (Ca_V(N)_) via the PTX-sensitive Gi/o protein pathway but without the involvement of AC or cAMP [166]. The significance of this modulation is related to the role of Ca_V(N)_ in the regulation of neurotransmitter release from nerve terminals, that is, somatostatin might reduce glutamate release from the principal hippocampal neurons. Electrophysiological studies on rat hippocampal slices have demonstrated that somatostatin reduces both AMPA and NMDA receptor-mediated excitatory postsynaptic currents (EPSCs) but does not affect GABA-A or GABA-B receptor-mediated inhibitory post-synaptic currents (IPSCs) in CA1 pyramidal neurons [167]. Pharmacological maneuvers suggested that the effect on glutamatergic currents is mediated by Gi/o protein through presynaptic mechanisms at CA1 Schaeffer collateral synapses. Also, in mouse hippocampal slices, somatostatin significantly inhibits both AMPA and NMDA EPSCs in CA1 pyramidal neurons; however, in this preparation, somatostatin proved to significantly increase GABA_A_ IPSCs [168]. A patch-clamp study on autapses formed by hippocampal neurons grown in isolation provided further evidence to support the role of somatostatin in inhibiting AMPA and NMDA excitatory transmission [169]. In particular, somatostatin and its analogues, seglitide and octreotide, reduced voltage-gated Ca^2+^ currents through the activation of presynaptic receptors and via PTX-sensitive G proteins. Definitive evidence for the role of somatostatin in inducing the presynaptic inhibition of glutamate release via SST_1_ was provided by Bagnoli’s laboratory. By using SST_1_-KO mice and selective pharmacological tools, they demonstrated the presynaptic localization of SST_1_ and the inhibitory effect of its activation on both AMPA/NMDA responses and glutamate release [99]. They also found that SST_1_ activation does not affect GABAergic inhibitory transmission. Other findings have provided evidence for the involvement of SST_2_ in inhibiting excitatory transmission at the synapse between the Schaffer collateral and CA1 pyramidal neuron in rat hippocampal slices [170]. The in vitro firing rate of these neurons was reduced by the application of octreotide without any significant effect on membrane potential [170]. In a nutshell, all of these data indicate that somatostatin can reduce firing activity by attenuating excitatory synaptic transmission. It is worth noting that somatostatin inhibits dendritic Ca^2+^ spikes by affecting N-type Ca^2+^ currents in DG granule cells [171]. Since these channels play an important role in signal processing in dendritic trees [172,173,174], somatostatin again stands out as a neuropeptide that is able to reduce neuronal excitability at multiple levels.

As a whole, the available data suggest that somatostatin depresses the activity of hippocampal pyramidal neurons by acting through pre- and post-synaptic mechanisms. At the presynaptic level, somatostatin reduces the release of glutamate, an excitatory neurotransmitter. In term of synaptic integration in the postsynaptic neurons, this favors inhibitory input, which, in turn, leads to a reduction in firing frequency. At the postsynaptic level, somatostatin stabilizes the membrane resting potential and tends to produce damping of depolarizations. In this way, pre- and post-synaptic effects of somatostatin act synergistically to reduce the excitability and to induce spike frequency adaptation. It is worth noting that there is no evidence for an effect of somatostatin on voltage-gated sodium channels in hippocampal pyramidal neurons. Thus, as in the retina, somatostatin targets firing properties, that is, it does not impede neurons to communicate, but it modulates how neurons communicate.

Although most of the available information on the activity of somatostatin in the hippocampus has been obtained by studying CA1 pyramidal neurons, there are reports suggesting that similar mechanisms might also operate in the CA3 region, although the intensity of the effects may vary somewhat [175], likely reflecting different expression level of SSTs (e.g., [176,177]). However, there are also remarkable differences among subfields. For example, unlike rat CA1 pyramidal neurons, somatostatin does not exert any postsynaptic effect on K^+^ currents in mouse DG granule cells and does not affect their firing properties [171]. Variations in somatostatin action across hippocampal regions might be related to their specific, local computational tasks. In guinea pig hippocampal slices, somatostatin augments LTP at mossy fiber/CA3 neuron synapses but has no effect on LTP in the Schaffer collateral/CA1 pathway [178]. In mouse DG, somatostatin depresses LTP at perforant path fiber/DG granule cell synapses [171]. It is also possible, as suggested by Baratta et al. (2002), that functional differences in somatostatin action between hippocampal subfields may be related to the specific subcellular localization of SSTs [171]. Finally, species-specific differences may also be responsible for this variability. For example, unlike results from the rat hippocampus, in mice, somatostatin does not affect EPSCs in CA1 pyramidal neurons [106]. Table 2 provides a summary of the main effects of somatostatin on ion channels in the principal neurons along the information transmission line DG → CA3 → CA1. It is interesting to note that, in the hippocampus, somatostatin also tends to attenuate neuronal communication by depressing membrane excitability through K^+^ channels and glutamate release through Ca^2+^ channels. Therefore, as proposed for retinal circuitry, it is conceivable that somatostatin plays a key role in setting the operational dynamic range of the hippocampal principal neurons.

## 5. Retina and Hippocampus: Common Themes

**Figure 7:**
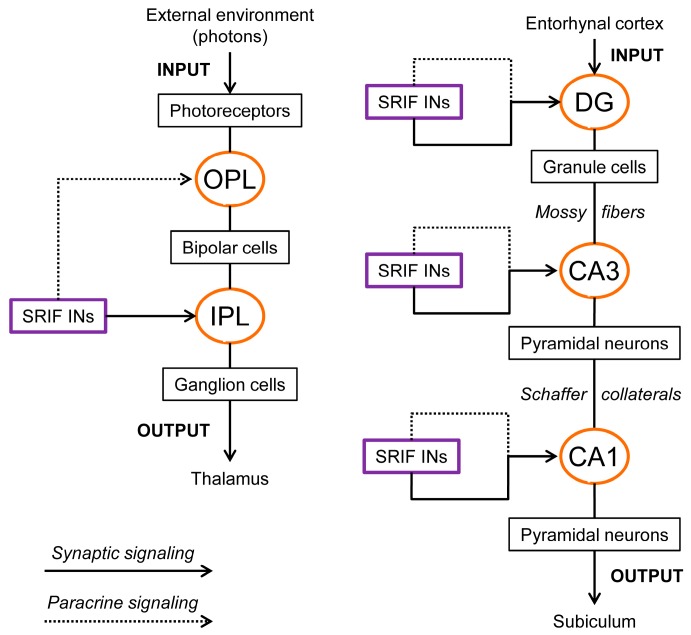
Schematic drawings of the retinal (left) and hippocampal (right) circuitries. The principal neurons form a main line of information flow with synaptic stations (orange circles) in specific zones of the anatomical formation. Somatostatinergic interneurons (SRIF INs) form parallel microcircuitries that control the information transfer between principal neurons. In the retina, SRIF INs are all located near the IPL and have extensive arborizations spanning horizontally. However, somatostatin can reach the OPL through diffusion (paracrine signaling), thanks to the compact organization of the retinal layers that keeps the two synaptic stations close by. In the hippocampus, on the contrary, the axons of the principal cells have to cross considerable distances to reach next synaptic station. Consequently, somatostatinergic interneurons occur in each hippocampal subfield, where they form local microcircuitries. Based on the synaptology of hippocampal SRIF INs and on the wide distribution of somatostatin receptors through all subcellular districts of principal cells, it is conceivable that a diffuse action (paracrine signaling) may also occur in addition to synaptic signaling.

In terms of structural organization, the retina and hippocampus differ significantly in terms of the design of the somatostatin-based system (see Section 3). This clearly reflects both anatomical constraints and the size of the tissue containing the circuitry. The retina is a flattened structure, which is ~300 μm thick in humans [179], with principal neurons densely packed in definite layers (Figure 1a). Somatostatinergic interneurons have cell bodies confined either in the INL or GCL (“displaced” amacrine cells), but they can easily affect both synaptic stations (IPL and OPL; Figure 7). The hippocampus, on the contrary, shows a typical convoluted organization, with principal neurons arranged in layers (as in the retina) and connected through specific bundles of nerve fibers (mossy fibers and Schaffer collaterals) (Figure 2a). The main synaptic stations (DG, CA3, CA1) are set apart, and therefore, somatostatinergic interneurons occur in each hippocampal subfield (Figure 7). Nonetheless, there are features of somatostatin-based signaling shared by both circuitries, suggesting that the conserved motifs of somatostatin activity are not related per se to the circuitry cytoarchitectonics.

### 5.1. Co-Transmission of GABA and Somatostatin

In both the retina and hippocampus, somatostatinergic interneurons are inhibitory neurons that provide mainly a local, parallel pathway that regulates the information flow through principal neurons. Somatostatinergic interneurons contain both GABA (a classical neurotransmitter) and somatostatin (a neuropeptide), and therefore, can affect target neurons in different but complementary ways [38,39,40]. GABA induces postsynaptic events that add up to other synaptic inputs. Somatostatin, on the contrary, reduces the release of the excitatory neurotransmitter glutamate via presynaptic effects (see Section 4) [99,116,142,167,169]. Therefore, it attenuates the excitatory postsynaptic events before they combine with the GABA-mediated ones in the process of synaptic integration. In other words, somatostatin facilitates the inhibitory action of the classical neurotransmitter GABA [167]. However, there are several aspects of co-transmission that require keen analysis to fully understand their impacts on synaptic communication [40,41,121,180]. Although somatostatin, like GABA, is released through a calcium-dependent mechanism and depends on action potential discharge [118,119,120], it is not clear how the activity level (i.e., firing frequency) affects the release of the two transmitter substances differentially. While classical neurotransmitter release is fairly irrespective of the action potential frequency, which mainly affects the postsynaptic response amplitude through synaptic integration, neuropeptide release requires bursting or high frequency activity [39,40,121]. Hippocampal O-LMs seem to possess autorhythmic activity [93,94], but the low frequency (about 7 Hz) of spontaneous firing is likely insufficient to drive somatostatin release [38,40]. Indeed, there are indications that somatostatin is released under conditions of enhanced electrical activity of the hippocampal neurons [119,120,171]. It is thus conceivable that somatostatin activity might be more important when the firing frequency is high. Under these conditions, somatostatin potentiates the GABA effect [40,41] and contributes to setting a cutoff frequency for firing in principal neurons, thus avoiding hyper-excitation. Thanks to the paracrine signaling mechanism, somatostatin is able to reach neighboring cells to modulate their activity (see Section 3.4). In this way, co-transmission allows somatostatin-GABAergic neurons to exert both point-to-point control of postsynaptic neurons as well as more diffuse control of neuronal microcircuitries [121]. In this regard, it is noteworthy that while classical neurotransmitters are released at synaptic sites, neuropeptides are released mostly from extra-synaptic sites (dendrites and axons) [39,40,121]. Although our understanding of GABA-somatostatin co-transmission in retinal amacrine cells is still limited, it is likely that the general mechanisms outlined above also applied to the retinal circuitry. The main difference between the hippocampus and the retina is due to the synaptology of somatostatinergic interneurons (Section 3). While O-LM interneurons make synaptic contacts with principal neurons (Figure 2b), somatostatinergic amacrine cells interact through conventional synapses mainly with other amacrine cells [62]. It is likely that GABA-somatostatin co-transmission may target separate yet interconnected subsets of neurons in the retinal circuitry, with the goal of setting the activity level of principal neurons. In this regard, it is interesting to note that recent data indicate that somatostatinergic amacrine cells target both dopaminergic amacrine cells and ganglion cells in a specific visual microcircuit [67].

### 5.2. Modulation of Ion Channels

Our current understanding of the effect of somatostatin on ion channels in both retinal and hippocampal circuitries underscores the role of K^+^ channels as molecular targets (Table 3). These channels control membrane excitability by counteracting depolarizing currents [157]. The functional goal is to reduce the probability of reaching the firing threshold, and, once spike discharge has begun, to facilitate the adaptation of the action potential frequency. An intriguing aspect of this regulation is the molecular diversity of K^+^ channels targeted by somatostatin (Table 1 and Table 2). Although it is possible to roughly distinguish two main subgroups, that is, channels controlling the resting potential (K_L_ and GIRK) and channels controlling firing (K_V_, K_M_, K_Ca_), this heterogeneity seems quite odd. A possible explanation can be envisaged by considering that the variety of K^+^ channels plays a key role in defining the discharge properties of different neurons [181] and also in regulating the electrical activity of dendrites [165]. Thus, it is reasonable to conceive that the target for somatostatin is “the K^+^ channel”, even though its molecular and biophysical identity may vary considerably according to the electrical signaling produced by specific neurons. Clearly, by affecting K^+^ channels, somatostatin influences neuronal communication and therefore reduces the level of network activity.

Another common motif of somatostatin activity is the inhibitory effect on voltage-gated Ca^2+^ channels (Table 3). The significance of this modulation is to produce a reduction in the presynaptic release of glutamate, the excitatory neurotransmitter in the main line of information transfer in both the retina and hippocampus. In other words, somatostatin tends to depress glutamatergic transmission. The outcome is again a “braking” effect on network activity. Although voltage-gated Ca^2+^ channels may exhibit molecular and biophysical heterogeneity across different neuronal types (Table 1 and Table 2), it worth mentioning that both Ca_V(L)_ and Ca_V(N)_ are high-voltage activated calcium channels, which are typically involved in regulating calcium-dependent neurotransmission [182].

On the basis of the widespread distribution of SSTs and the effects on ion channels, it is conceivable that somatostatin does not mediate a point-to-point action, but rather, it affects the excitability of the whole network “globally”. The available data suggest that, at least in the hippocampus, somatostatin release is enhanced under conditions of increased activity [119,120]. Thus, it is tempting to speculate that somatostatin impedes principal neurons from becoming over-responsive by reducing their excitability with consequent damping of the firing rate. In other words, somatostatin works as homeostatic regulator of network activity.

### 5.3. The Logic of Somatostatin-Based Signaling

The retina and hippocampus differ in terms of their specific functional goals. Visual stimuli are processed by the retina in a rather complex way to make the firing of the ganglion cells informative (sensory coding) for the thalamus (e.g., [22,137,183,184,185]). The hippocampus, on the other hand, is a circuitry organized to handle highly-processed sensory information from other brain circuits and to play roles in learning and memory (e.g., [186,187,188,189,190]). Despite these differences in computational tasks, both the retina and hippocampus rely on somatostatin to assure the proper functioning of their circuitries. This is obtained through common molecular and cellular mechanisms that lead to a “braking” effect on the activity of principal neurons. Thus, the somatostatinergic system works as a network stabilizer. Several experimental and clinical observations support this notion. In the rabbit retina, somatostatin acts on the whole retinal circuitry to improve the ganglion cell output over a long-lasting timescale, thus assuring stability in visual processing [124]. In SST_1_-KO mice, the retinal level of somatostatin increases, and SST_2_ becomes overexpressed [70]. As a consequence of this “disturbance” in the somatostatinergic system, electroretinograms (ERGs) recorded in response to light flashes display altered oscillatory potentials (OPs) [191]. Since OPs are likely generated by bipolar cells [192], OP alterations suggest a malfunctioning of the vertical circuit of information flow (Figure 1b) in the presence of disrupted somatostatin signaling. It is noteworthy that somatostatinergic amacrine cells represent only a small percentage of the whole retinal cell population [61,62], yet disruption in their signaling affects network functionality. Evidence for a role of somatostatin in setting the level of neuronal activity irrespective of visual processing was provided by Vuong et al. (2015). In the mouse retina, somatostatin plays a compensatory role in a non-image-forming visual circuit involving DA amacrine cells and M1 ipRGCs: by adjusting the retinal DA level, somatostatin assures the stability of the light response by M1 ipRGCs, avoiding excessive firing [67]. The role of somatostatin in the etiopathogenesis of epilepsy is a further example of how this peptide is required to maintain network stability. It is well known, in fact, that the loss of somatostatinergic interneurons in the dentate gyrus contributes to epileptogenesis, and the administration of somatostatin has shown antiepileptic properties [193]. Electrophysiological studies on in vitro models of epilepsy have provided evidence that somatostatin acts in CA1 and CA3 to reduce hyper-excitability [175]. In hippocampal CA1 pyramidal neurons, spontaneous epileptiform bursting is significantly reduced by somatostatin through the activation of SST_4_ coupled to an increase in the M-current [106]. In a nutshell, somatostatin exerts a “braking” action on the functioning of network principal neurons; by exploiting its effects on membrane excitability and neurotransmission, and due to its property of being a diffusible substance (paracrine signaling; Table 3), somatostatin creates a microenvironment inside the network that drives neurons to work under “low-pass filter” conditions in terms of their firing frequency.

Somatostatinergic neurons are not the only interneurons capable of exerting inhibitory control. In the retina, both horizontal cells and amacrine cells mediate feedback and feedforward inhibition in OPL and IPL [12,30,194]. In addition, many different types of amacrine cells occur in the mammalian retina, adding further complexity to the inhibitory system [12,37]. In the hippocampus, GABAergic interneurons are remarkably diverse, both morphologically and physiologically [44,45,195]. Besides their structural properties and anatomical organization, an amazing feature of inhibitory interneurons in both the retina and hippocampus is their neurochemical heterogeneity, which depends mostly on the presence of a second “modulatory” transmitter in addition to the classical fast inhibitory neurotransmitters (GABA or glycine). In the retina, several neuropeptides have been identified in subsets of amacrine cells including substance P, neuropeptide Y (NPY), vasoactive intestinal peptide (VIP), and cholecystokinin (CCK), just to mention a few [32,196]. Also, hippocampal interneurons may express NPY, CCK, and VIP [44]. Like somatostatin, but unlike fast neurotransmitters, neuropeptides act over a slow timescale and a wider space outside the synaptic cleft [39,40,121]. Why do neural networks require so many diverse inhibitory interneurons, and why do they rely on synaptic mechanisms operating on complementary timeframes (co-transmission)? To explain this riddle, we have to consider that inhibition is fundamental for the production of specific computational tasks characterizing different neural networks. In the retina, lateral information flow is controlled by inhibitory interneurons (horizontal cells and amacrine cells) to allow visual processing to occur. For example, horizontal cells play a key role in establishing center-surround organization to the receptive fields of bipolar cells by producing surrounding inhibition in the OPL. These receptive fields are further shaped in terms of both spatial and temporal characteristics by the activity of amacrine cells, which are key regulators of ganglion cell activity (e.g., [12,30]). In the hippocampus, GABAergic interneurons control synaptic integration and spike initiation in pyramidal cells, thus shaping the timing of afferent and efferent information flow. In addition, they produce synchronization of local cortical circuits; in short, inhibitory interneurons affect the network dynamics [17,44,197]. Thus, the remarkable anatomical, neurochemical, and physiological diversity of inhibitory interneurons matches the complexity of network functioning because they are required for several computational tasks.

**Figure 8:**
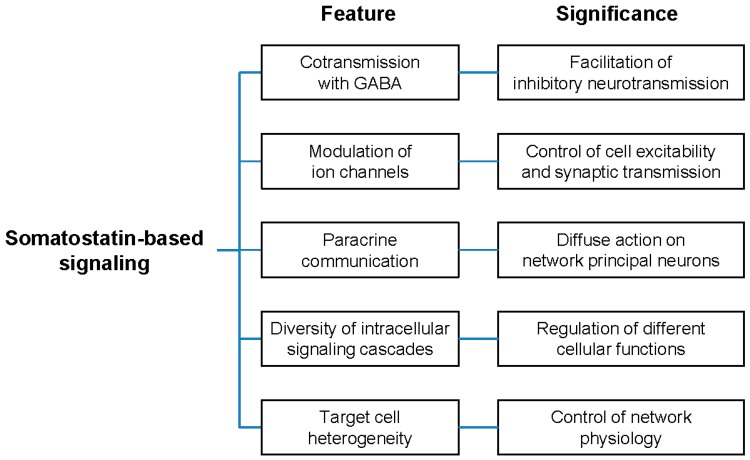
The complexity of somatostatin-based signaling in the retina and hippocampus.

Given the great variety of inhibitory interneurons, what is so special about somatostatinergic interneurons? Although basically all inhibitory interneurons share the property of braking the excessive activity of principal cells, we are tempted to speculate that somatostatin may represent a unique modulatory substance that is able to affect not only network dynamics but also network physiology. In this review, we have addressed the role of somatostatin as a regulator of information transmission between neurons. However, somatostatin receptors are also expressed by other cellular types in the same tissue housing the neuronal network. For example, somatostatin-binding sites as well as SST mRNAs have been detected in astrocytes [198,199,200], suggesting that somatostatin may exert biological actions on these glial cells. Indeed, pharmacological assays have shown that somatostatin inhibits endozepine release in cultured rat astrocytes through the activation of SST_1_, SST_2_, and SST_4_ negatively coupled to the canonical transduction pathway AC/cAMP/PKA [201]. Also, a recent paper by Carmignoto’s laboratory [202] provided compelling evidence that somatostatin-expressing interneurons affect intracellular Ca^2+^ signaling through SST_4_ in adult cortical astrocytes. Glia-neuron crosstalk plays a key role in nervous tissue physiology, being involved in diverse processes, such as volume regulation, metabolism, and information flow [203,204]. Thus, findings on the effect of somatostatin on astrocytes underscore the importance of this neuropeptide for the general functioning of the network. It is worth noting that fibers of the predominant glial cell in the retina, the Müller cell, have been reported to be immunoreactive for SST_1_ and SST_2_ [69]. SSTs, in particular, SST_2_, also occur in the endothelial cells of blood vessels [2] and exert antiangiogenic effects, preventing pathological neovascularization in several experimental models (e.g., [205,206,207,208,209]). Somatostatin has been proven to counteract the dysregulation of pro-angiogenic factors that characterize diabetic retinopathy [63,196,210]. The involvement of somatostatin in so many diverse processes, such as the regulation of astrocyte functioning and vascularization processes, is understandable by considering that this neuropeptide works through the activation of different receptors (SST_1–5_), which, in turn, are coupled to different signaling pathways. The diversification of the intracellular signaling cascades is responsible for “the pleiotropic cellular functions” of somatostatin [115]. In summary, experimental observations suggest that somatostatin released by somatostatinergic interneurons is important not only for the correct functioning of the neuronal network, but also for the maintenance of the milieu in which neurons work (Figure 8).

While somatostatin is a key molecule that is able to control many diverse aspects of neuronal tissue physiology, clearly, it cannot regulate all of the cellular processes underlying the complex functioning of a living tissue. Indeed, other peptidergic systems are known to operate in neural networks. For example, an NPY-based system occurs both in the retina [211] and the hippocampus [212]. The available data support the notion that neuropeptidergic systems work as neurochemical devices that complement and balance each other’s actions [213]. In the developing retina, for example, neuropeptides play a role not only in regulating network growth but also in modulating network activity according to specific timeframes before and after eye opening [214]. To make the matter even more complicated, neuropeptides may also co-localize in the same interneurons. For instance, many GABAergic neurons contain both somatostatin and NPY in hippocampus DG [212]. Understanding how peptidergic systems coordinate their activities to regulate network functioning represents an unsolved yet exciting issue in the field of neuroscience [121,215].

## 6. The Role of Somatostatin in Network Functioning: Implications for Health and Disease

After several years of studies, there is now overwhelming evidence that somatostatin plays a key role in the functioning of retinal and hippocampal networks. By modulating membrane excitability and neurotransmitter release, somatostatin contributes significantly to setting the level of activity of principal neurons. The occurrence of somatostatin receptors in different cellular types further underscores the importance of this neuropeptide, not only in the regulation of neuron activity but also in the general physiology of the tissue housing neural networks. Indeed, disturbance in somatostatin signaling is a hallmark of many brain diseases, including disorders of the retina and hippocampus (e.g., [8,44,63,77,115,193,196,216,217,218]). With this premise, it is not surprising that the somatostatinergic system has attracted the interest of researchers aimed at finding adequate therapeutic solutions for debilitating diseases such as diabetic retinopathy and epilepsy.

In the retina, there is increasing evidence that somatostatin may act as a neuroprotective agent against ischemic insults, although the mechanism underlying its protecting effect is far from being clarified [63,77,196,219]. Several observations suggest that somatostatin may ameliorate neurodegeneration consequent to ischemia by inhibiting glutamate release in the retinal circuitry, an event that would occur through presynaptic SST_2_ [116]. Further support for the involvement of SST_2_ in mediating somatostatin neuroprotective activity has been provided by studies on SST-KO retinas. The retinal response to ischemia is significantly spared in the presence of SST_2_ overexpression, as in SST_1_-KO animals, whereas it is drastically increased when SST_2_ has been knocked out [191,220,221]. Somatostatin has been also shown to avoid high glucose-associated neurodegeneration in the retina, as demonstrated in rat models of diabetic retinopathy, in which somatostatin eye drops prevented functional abnormalities (as assessed by ERG) and inhibited glutamate accumulation [222]. More recently, somatostatin topical administration was tested in clinical trials and proved useful in avoiding the progression of pre-existing retinal neurodysfunction [223].

In the hippocampus, several studies have provided evidence for a role of somatostatin in depressing the neuronal hyper-excitability underlying seizures (e.g., [106,175]). Direct injection of somatostatin into the hippocampus produced a strong inhibitory effect on behavioral and electrical seizures recorded in vivo [216]. Alterations in the somatostatinergic system have been documented in virtually all experimental models of temporal lobe epilepsy (TLE) [96,193,216,224]. A loss of neurons expressing somatostatin has been demonstrated in the hippocampus of patients with TLE [225,226]. Nonetheless, information on the contribution of specific SSTs on the somatostatin-mediated inhibition of epileptiform activity is still controversial and apparently species-dependent. In mice, SST_2_ does not affect kainate seizure [227], whereas the activation of SST_1_ plays a role in reducing hippocampal hyper-excitability [117]. However, additional results have demonstrated the requirement for a critical SST_2_ density to regulate hippocampal activity. Indeed, SST_2_ upregulation as a consequence of somatostatin gene deletion (SRIF-KO mice) is able to control hippocampal bursting and excitatory transmission [168]. On the other hand, data on rats seem to exclude the idea that SST_1_ mediates the in vivo anticonvulsive effects of somatostatin [100]; rather, SST_2_ is likely to be involved (e.g., [170,228]). Knocking out SST_4_ strongly affects epileptiform activity in the mouse CA1 subfield, although also other receptors (SST_2_, SST_3_) may play roles [106]). Finally, there are indications for functional cooperation between rat hippocampal SST_3_/SST_4_ and SST_2_ in mediating anticonvulsive effects [229]. As a whole, these studies suggest that the anticonvulsant action of somatostatin may be mediated by species-specific receptors. Improving our understanding of how the somatostatin receptor network works, especially in the human hippocampus, is clearly a pre-requisite for designing new drugs with anti-epileptic activity [230].

## 7. Conclusions and Future Perspectives

Somatostatinergic interneurons are key components of the signaling systems inside the retina and hippocampus, as they fulfill a modulatory role in determining the activity levels of principal neurons. This is obtained through somatostatin, which regulates single cell excitability and neurotransmission. By exploiting its capability to work as both a synaptic and paracrine substance, somatostatin can affect the functioning of the whole neural network, and it helps to shape the firing dynamic range of the output neurons. In particular, somatostatin sets the cutoff frequency in spiking neurons to avoid dangerous hyper-excitation. Besides its involvement in regulating network operation, somatostatin also modulates other biological processes by affecting the functions of non-neuronal cells, such as glial cells and endothelial cells. Thus, somatostatin contributes to the determination of homeostasis in neural networks. Since somatostatinergic interneurons are widespread throughout the brain, we believe that our reasoning on the retina and hippocampus may also apply to other neuronal circuits. Specifically, somatostatin creates an adequate microenvironment that allows neurons to operate correctly. In this regard, the transgenic somatostatin-Cre mouse strain, which exhibits a low level of endogenous somatostatin, may represent a promising model to gain further insights into the role of somatostatin-based signaling in the regulation of neural network functioning.

Despite several years of investigation, our understanding of the molecular and cellular physiology of somatostatinergic system in neural networks is far from complete. Somatostatin function has been addressed through a number of experimental strategies, including pharmacological tools directed at somatostatin receptors, and transgenic mouse models with knocked-out receptors. These approaches, however, pose several concerns. Ligand selectivity and the somatostatin receptor involved may influence the efficiency of coupling to intracellular events, questioning their physiological relevance. Furthermore, functional cooperation between different receptor subtypes may complicate the interpretation of the observed effects. Finally, compensatory expression of other receptor subtypes may occur when knocking out a given somatostatin receptor. In short, molecular mechanisms underlying receptor–receptor interactions, receptor subtype-selective signaling in target cells, and their impact on network physiology are only partially understood. This represents a promising avenue for the development of specific pharmacological SST modulators that are ideal candidates to counteract neuronal diseases by exerting neuroprotective actions.

## Figures and Tables

**Table 1:** Ion channels and their modulation by somatostatin (↑: potentiation; ↓: reduction; = no effect) in the retinal cells of the information transmission line RP → RBC → GC.

Ion Channels	Rod Photoreceptor	Rod Bipolar Cell	Ganglion Cell
Na_V_	*	*	=
Ca_V(L)_	↓	↓	↓
K_V_	↑	=	↑
K_Ca_	=	↓	=

*: not present; Ca_V(L)_: voltage-gated L-type calcium channel; K_Ca_: voltage-gated and Ca^2+^-dependent potassium channel; K_V_: voltage-gated potassium channel; Na_V_: voltage-gated sodium channel.

**Table 2:** Ion channels and their modulation by somatostatin (↑: potentiation; ↓: reduction; = no effect) in the principal hippocampal neurons of the information line DG → CA3 → CA1.

Ion Channels	DG Granule Cells	CA3 Pyramidal Neurons	CA1 Pyramidal Neurons
Na_V_	=	=	=
Ca_V(N)_	↓		↓
K_M_, K_L_, GIRK	=	↑ *	↑

Ca_V(N)_: voltage-gated N-type calcium channel; GIRK: G-protein gated, inwardly rectifying potassium channel; K_L_: voltage-insensitive, leak potassium channel; K_M_: non-inactivating, voltage-dependent potassium channel; Na_V_: voltage-gated sodium channel. *: somatostatin causes membrane hyperpolarization, but the identity of K^+^ channels is currently unknown.

**Table 3:** The somatostatinergic system in the retina and hippocampus: shared properties and mechanisms (↑: potentiation; ↓: reduction) along the main line of information transfer (retina: RP → RBC → GC; hippocampus: DG → CA3 → CA1).

		Retina	Hippocampus	Significance
SRIF interneuron	Co-transmitter	GABA	GABA	Fast synaptic inhibition in microcircuits
	Autoreceptor	SST_1_	SST_1_	Control of somatostatin secretion
SRIF receptors	Distribution	Wide	Wide	Diffuse (over several μm) and global action
	Presynaptic neurons	SST_2_	SST_1_/SST_2_	Targets calcium channels
	Output neurons *	SST_4_	SST_4_	Targets potassium channels
SRIF action on glutamatergic neurotransmission	Presynaptic site	Ca^2+^ channels (↓)	Ca^2+^ channels (↓)	↓ neurotransmitter release
	Postsynaptic site	K^+^ channels (↑)	K^+^ channels (↑)	↓ firing rate
SRIF signaling	Global action	Inhibitory	Inhibitory	Network stabilizer

SRIF: somatostatin; SST: somatostatin receptor. *: retinal ganglion cells and CA1 pyramidal cells.

## Abbreviations

4-AP	4-amino pyridine
AC	Adenylyl cyclase
AMPA	α-amino-3-hydroxy-5-methyl-4-isoxazolepropionic acid
BiC	Bistratified cell
cAMP	Cyclic adenosine monophosphate
CA	Cornu ammonis
Ca_V(L)_	Voltage-gated L-type calcium channel
Ca_V(N)_	Voltage-gated N-type calcium channel
CCK	Cholecystokinin
CNS	Central nervous system
DA	Dopamine
DG	Dentate gyrus
EC	Entorhinal cortex
EPSC	Excitatory post-synaptic current
EPSP	Excitatory post-synaptic potential
ERG	Electroretinogram
GABA	γ-aminobutyric acid
GC	Ganglion cell
GCL	Ganglion cell layer
GIRK	G-protein activated, inwardly rectifying potassium channel
GPCR	G-protein coupled receptor
INL	Inner nuclear layer
IPL	Inner plexiform layer
IPSC	Inhibitory post-synaptic current
K_Ca_	Voltage-gated and Ca^2+^-dependent potassium channel
KO	Knock-out
K_V_	Voltage-gated potassium channel
LTP	Long-term potentiation
M1 ipRGCs	Melanopsin-containing, intrinsically photosensitive retinal ganglion cells
Na_V_	Voltage-gated sodium channel
NMDA	N-methyl-D-aspartate
NPY	Neuropeptide Y
O-LM	Oriens lacunosum-moleculare interneuron
ONL	Outer nuclear layer
OPL	Outer plexiform layer
PKA	Protein kinase A
PKC	Protein kinase C
PP	Perforant pathway
PTX	Pertussis toxin
RBC	Rod bipolar cell
RP	Rod photoreceptor
s.l.m.	Stratum lacumosum-moleculare
s.o.	Stratum oriens
s.p.	Stratum pyramidale
s.r.	Stratum radiatum
SRIF	Somatotropin release-inhibiting factor
SST	Somatostatin receptor
TLE	Temporal lobe epilepsy
VIP	Vasoactive intestinal peptide
